# A Long-Term Study on the Bactericidal Effect of ZrN-Cu Nanostructured Coatings Deposited by an Industrial Physical Vapor Deposition System

**DOI:** 10.3390/nano14060496

**Published:** 2024-03-10

**Authors:** Sahand Behrangi, Eva Staňková, Ivo Sedláček, Lucie Šimoníková, Pavel Souček, Vilma Buršíková, Vjačeslav Sochora, Karel Novotný, Petr Vašina

**Affiliations:** 1 Department of Plasma Physics and Technology, Faculty of Science, Masaryk University, Kotlářská 2, 61137 Brno, Czech Republic; soucek@mail.muni.cz (P.S.); vilmab@physics.muni.cz (V.B.); vasina@physics.muni.cz (P.V.); 2 Czech Collection of Microorganisms, Department of Experimental Biology, Faculty of Science, Masaryk University, Kamenice 5, 62500 Brno, Czech Republic; evickakroupova@seznam.cz (E.S.); ivo@sci.muni.cz (I.S.); 3 Department of Chemistry, Faculty of Science, Masaryk University, Kamenice 5, 62500 Brno, Czech Republic; lucies@mail.muni.cz (L.Š.); codl@sci.muni.cz (K.N.); 4 SHM, s.r.o., Průmyslová 3020/3, 78701 Šumperk, Czech Republic; sochora@shm-cz.cz

**Keywords:** magnetron sputtering, *Escherichia coli*, door handles, bactericidal efficiency, ZrN-Cu coating

## Abstract

ZrN-Cu coatings containing two different amounts of Cu (~11 at.% and ~25 at.%) were deposited using an industrial physical vapor deposition (PVD) system. The as-deposited coatings exhibited 100% bactericidal efficiency against *Escherichia coli* CCM 3988 for an exposure time of 40 min. Subsequently, the samples were attached onto our faculty’s door handles for six months to study the coatings’ long-term effectiveness and durability under actual operational conditions. The samples were periodically evaluated and it was observed that the coatings with 25 at.% Cu performed better than the ones with 11 at.% Cu. For example, following 15 days of being touched, the bactericidal effectiveness of the sample containing 25 at.% Cu dropped to 65% while it fell to 42% for the sample containing 11 at.%. After 6 months, however, both samples showed bactericidal efficiency of ~16–20%. The bactericidal efficiency of the samples touched for 6 months was successfully restored by polishing them. Furthermore, a group of samples was kept untouched and was also evaluated. The untouched samples with Cu content of ~25 at.% did not show any drop in their bactericidal properties after 6 months. ZrN-Cu coatings were concluded to be promising materials for self-sanitizing application on high-touch surfaces.

## 1. Introduction

Pathogen transmissions through contaminated common touch surfaces (fomites) are a major cause of hospital-acquired infections or healthcare-associated infections (HAIs) [1,2]. HAIs have been proven to cause high morbidity and mortality rates and impose an enormous economic burden on healthcare systems worldwide [1,3,4]. In addition to healthcare centers, community gathering centers such as public transportation, educational institutions, catering establishments, sports facilities, and hotels are highly susceptible to pathogenic microbes being spread [5,6].

It has been observed that respiratory droplets containing pathogens like bacteria can land on surfaces and consequently be mechanically transmitted to those who touch such surfaces. Depending on various factors such as temperature and relative humidity, some pathogens might survive on high-touch surfaces for days, weeks, or even months. Thus, developing self-sanitizing coatings on high-touch surfaces seems a practical solution to this threat, by providing a means of reducing the spread of pathogenic microorganisms [2,7,8,9,10,11].

Copper (Cu) and silver (Ag) are the two best known metals demonstrating high antimicrobial activity [12,13]. Generally, Cu has been shown to have superiority over Ag for the following reasons: (i) unlike Ag, Cu is an essential element for the human body [14]; (ii) the human body can metabolize Cu, while Ag tends to remain unmetabolized for longer [15,16]; (iii) Ag shows higher toxicity than Cu [17]; (iv) some studies have shown that Cu has a higher contact-killing efficiency against microorganisms [5,18,19]; and (v) using Cu is more cost-effective than using Ag [20]. The antimicrobial activity of Cu and its alloys against bacteria, viruses, and fungi has been well-established in recent decades [20,21,22,23,24,25,26]. 

Copper is a promising material for developing antibacterial surfaces. This is because copper exposure destroys plasmid DNA, which is the main mechanism for bacteria to transfer and acquire resistant genes. Moreover, the rapid bactericidal action of copper does not allow bacteria to multiply and mutate [27]. 

It might be thought that installing Cu-coated parts in hospitals would be so costly that it would not be worth doing. Intriguing work by Michels et al. [28] showed that installing such surfaces is reasonably priced compared to the treatment cost of HAIs. According to their assessments, the time taken to recoup the extra cost of Cu surface installation is only 37–44 days, which seems highly cost-effective.

The long-term antimicrobial activity of Cu and its alloys under actual operational conditions has been studied by some researchers [1,2,29,30,31] but still requires more investigation. In practical applications like high-touch surfaces, Cu alone would fail to function properly due to its low wear and corrosion resistance. Therefore, this study focuses on assessing the long-term bactericidal effectiveness of ZrN-Cu coatings on door handles. Door handles are prone to wear; hence, Cu (as a soft metal) must be co-deposited with a hard and wear-resistant material such as zirconium nitride (ZrN) to improve its durability and longevity. Furthermore, ZrN has shown to have a slight bactericidal effect [32,33,34].

The question that might arise here is why we should develop a coating instead of manufacturing the whole part out of copper alloy. Answering this question, it must be pointed out that magnetron sputtering is a highly versatile and cost-effective method for developing hard coatings on complex-shaped parts such as door handles. In contrast, manufacturing a whole body of a ZrN + Cu composite material would be a complex process due to the inherent differences in the properties of the ceramic and the metal phases. By utilizing magnetron sputtering to develop a hard yet antibacterial coating on the surface of the door handle, a more efficient and cost-effective solution can be achieved. This approach allows for the development of a durable and effective antibacterial coating while avoiding the challenges and costs associated with manufacturing a whole body of a composite material. 

In order to replicate a real-life scenario where frequently touched surfaces are exposed to bacteria, the bacterial exposure time was reduced to 40 min, as opposed to the standard 24 h testing period [35,36]. This adjustment was made to guarantee effective sanitization, particularly in public areas and on high-touch surfaces such as door handles. Door handles are susceptible to wear and the spread of pathogens. Therefore, applying a ZrN-Cu coating would fabricate a self-sanitizing surface with good mechanical properties that improve its lifespan.

Following our previous work, which demonstrated the outstanding bactericidal properties of ZrN-Cu coatings [37], we wanted to assess the long-term bactericidal activity of the coatings. In the present study, the samples were deposited by an industrial physical vapor deposition (PVD) system combining magnetron sputtering and arc evaporation methods, and were then attached onto door handles. The door handles were installed on different doors at our faculty to be regularly touched by students and staff. The samples were first removed after 15 days and, afterwards, monthly, to evaluate their bactericidal efficiency against *Escherichia coli* CCM 3988. A group of samples with identical chemical composition was kept untouched to study and determine the effect of natural aging on the samples. 

The study presents several novel findings that contribute significantly to the field of antibacterial coatings. The biggest novelty of this work is that it is not only devoted to the study of the properties of as-deposited coatings, but also to their long-term performance when the coatings are used in a real environment. Another notable novelty of this study is the use of an industrial scale magnetron sputtering machine to develop the coatings. This approach ensures that the coatings can be industrially developed and used in the future without the need for further experiments to upscale the process. Another unique aspect of this study is the use of two approaches to evaluate the antibacterial efficiency of the coatings. The study (1) applied a specific strain (*Escherichia coli* CCM 3988) on the samples according to ISO 22196 standard procedure [38] to see whether the antibacterial activity would change during 6 months, and (2) exposed the samples to all kinds of bacteria coming from human touch to observe whether there would be an overall change in the number of viable naturally occurring bacteria. Finally, unlike some other works, this study did not use any disinfectant to observe the natural changes in the antibacterial activity of the coatings. Together, these findings provide valuable insights into the development of antibacterial coatings and their testing in a real environment, and have significant implications for future research in this area.

## 2. Materials and Methods

### 2.1. Synthesis

All depositions were performed using an industrial deposition system at SHM s.r.o. Šumperk, Czech Republic. The internal dimensions of the deposition chamber were measured as 550 mm × 550 mm × 850 mm. A combination of arc evaporation (for the Zr target) and magnetron sputtering (for the Cu target) in a nitrogen atmosphere was utilized. The Zr target (99.2 wt.% Zr) and the Cu target (99.95 wt.% Cu) both had a cylindrical shape with a diameter of 96 mm and length of 445 mm. 

Three types of substrates were used for the depositions: (1) round coupons made of AISI D2 tool steel with a diameter of 20 mm; (2) cylindrical substrates made of AISI 304 stainless steel with the dimensions 112 mm × 21 mm; and 3) Si (100) wafers with the dimensions 15 mm × 15 mm. Pre-cleaning of the substrates was performed in an ultrasonic bath containing acetone and isopropanol. Prior to deposition, the substrates were subjected to Ti ion etching for a duration of 9 min using an Ar flow rate of 35 sccm. The etching process was carried out with a substrate bias voltage of −1000 V and at a temperature of 400 °C. Afterwards, an adhesion layer (ZrN) was deposited on the substrates with the parameters shown in Table 1, followed by ZrN-Cu deposition. Two magnetron power values of 1.2 kW and 2.2 kW were applied to obtain different copper contents. The other deposition parameters are presented in Table 1. The round coupons were coated from both sides for 100 min each. Once the deposition had been completed, the samples were cooled down to room temperature inside the chamber. 

### 2.2. Characterization

The analysis of the microstructure and measurement of the chemical composition of the samples were conducted using a Tescan Mira3 Scanning Electron Microscope (SEM, Tescan, Brno, Czech Republic) equipped with an Oxford Instruments X-Max^50^ X-ray Energy Dispersive Spectroscope (EDS, Oxford Instruments, Abingdon-on-Thames, UK). Quantification of the elemental composition was made using the internal standards library supplied by Oxford Instruments within the AZtec Software (Version 4.2, Oxford Instruments, Abingdon-on-Thames, UK).

To investigate the crystalline phase structure of the samples, a Rigaku SmartLab Type F X-ray Diffractometer (XRD, Rigaku Corporation, Tokyo, Japan) was utilized. The XRD equipment was set up in a Bragg–Brentano configuration with Cu Kα radiation (λ = 1.5418 Å). The size of the crystallites was calculated by applying the Scherrer formula (Equation (1)) to the XRD diffractograms.

(1) $$D=\frac{0.9\lambda }{B\cos\theta_{B}}$$

where *λ* is the wavelength of the X-ray beam used for the characterization, *B* is the full width at half maximum (FWHM) of the diffraction peak, and *Ɵ* is the Bragg angle [39].

The surface roughness of the coatings was measured using an Olympus Lext OLS4000 laser confocal microscope. The measurements were performed on 130 µm × 130 µm images. The roughness/waviness cut-off wavelength was set to 25 μm. The average arithmetical mean height (parameter *S_a_*) was selected to evaluate surface roughness. The hardness and elastic moduli of the coatings were measured using a Hysitron TI950 TriboIndenter (Bruker, Billerica, MA, USA) equipped with a Berkovich tip. The nanoindentation measurements were carried out by means of rapid mechanical property mapping (XPM) using a constant loading rate of 100 mN/s and maximum loads of 10 mN. The unloading segments were evaluated using the standard evaluation process based on the Oliver and Pharr model [40]. The mechanical property data were calculated as averages of 100 indentations (matrix 10 × 10; distance between individual indentations 5 µm; field size 50 × 50 µm^2^). At least two mechanical property maps were carried out at different places on the studied samples.

### 2.3. Evaluation of Long-Term Bactericidal Properties

**Figure 1:**
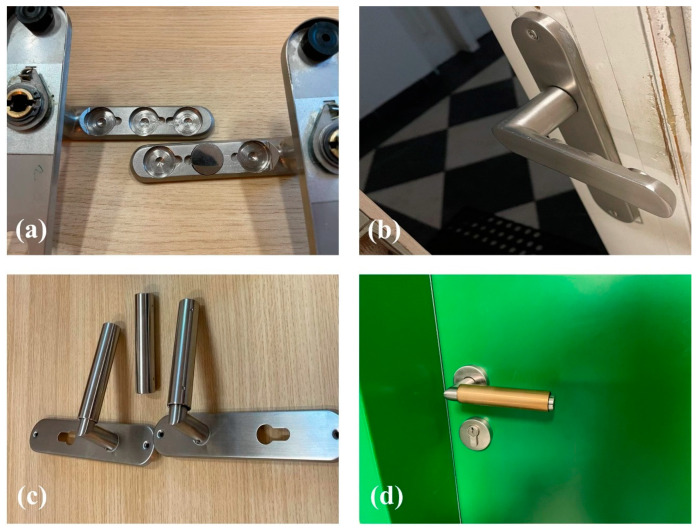
(**a**,**b**) The flat samples attached onto the door handles to evaluate the bactericidal efficiency against *E. coli* CCM 3988 over time, (**c**,**d**) cylindrical samples attached onto the door handles to assess the ability of the coatings to inhibit naturally occurring bacteria.

The coupons were attached onto the door handles of the Faculty of Science, Masaryk University, Brno, Czech Republic for 6 months, from May 2022 to October 2022, as shown in Figure 1a,b.

Eight samples were deposited for each Cu concentration—four were attached onto the door handles to study the effect of touch and four (with identical chemical composition) were kept untouched to study the aging effect. Both groups of samples were first evaluated after 15 days, and then monthly. Their bactericidal properties were assessed according to the procedure previously used [37], but with a slight change: the surface was exposed only to one bacterium (*E. coli* CCM 3988) and the exposure time was fixed at 40 min.

The cylindrical samples were attached onto the door handles of the Czech Collection of Microorganisms (CCM), Masaryk University, Brno, Czech Republic (Figure 1c,d). Two samples per Cu concentration were installed to evaluate the qualitative evolution of the bactericidal efficiency of the ZrN-Cu coatings. In this approach, instead of applying a specific bacterium to the surface, the overall viable bacteria naturally occurring on the surfaces were counted without distinguishing their type.

To assess the bactericidal performance of these samples the following steps were taken:

1. Obtaining the bacterial samples using a sterile cotton swab saturated with phosphate-buffered saline (PBS) along the whole length of the door handles (210 mm) twice. One side of the swab wiped the upper part of the handle, and the other side wiped the bottom part of the handle.
2. Putting the swab into a tube with 1 mL PBS.
3. Vortexing the tube with the swab thoroughly for 60 s.
4. Pipetting 100 µL of the solution after thorough vortexing on Tryptone Soya Agar (TSA) plates in triplicate (so 300 µL in total on 3 TSA plates).
5. Spreading the solution evenly across the whole plate using a spreader.
6. Incubating the TSA plates at 30 °C for 48 h.
7. Enumeration of the colony-forming units (CFU) grown.

### 2.4. Cu Release Measurement

The concentration of copper released from the ZrN-Cu coatings deposited on AISI D2 steel was measured using the inductively coupled plasma optical emission spectroscopy (ICP-OES) technique in Milli-Q water [13,16,41]. The ZrN-Cu coatings were immersed in Milli-Q water for 24–120 h. Following sample removal and prior to analysis, the aqueous solutions were acidified by adding 60 µL of concentrated HNO_3_ (65%, Analpure, Analytika, Czech Republic) to stabilize the Cu released in the Milli-Q water. Cu Certified Reference Material (CRM) (1000 ± 2 mg/L, Analytika, Czech Republic) was used for analysis. The solutions were analyzed with an ICP-OES spectrometer (iCAP PRO XPS, Thermo, Waltham, MA, USA) using the calibration line method. For the Cu measurements, emission lines Cu(I) 324.754 nm and Cu(I) 327.396 nm were used. The other measurement parameters were as following: axial observation; RF power: 1050 W; nebulizer gas flow: 0.50 L/min; auxiliary gas flow: 0.5 L/min; cool gas flow: 11.5 L/min; exposure time: 7 s; number of repetitions: 4.

## 3. Results and Discussion

### 3.1. Chemical Composition and Crystalline Structure

The chemical composition and mechanical properties of the ZrN-Cu samples deposited are summarized in Table 2. It was observed that the Cu content of the coatings was directly proportional to the power applied to the Cu target. Thus, samples with two different Cu contents were developed and labeled Cu11 and Cu25, based on the Cu content they contained. It should be mentioned that all samples contained 6–13 at.% of C and 5–8 at.% of O, corresponding with atmospheric contamination, as well as 0.3–0.4 at.% of Hf, which came from the Zr target. These were considered contaminants and were not taken into further consideration. 

**Figure 2:**
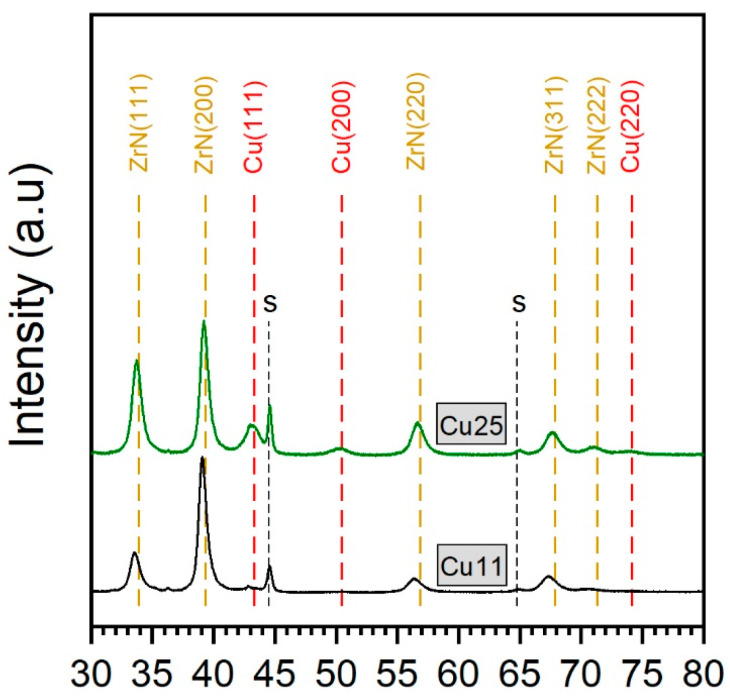
XRD patterns of ZrN-Cu coatings with two different copper contents. Si diffractions (denoted with S) come from the substrate.

Figure 2 displays the XRD diffractograms of the ZrN-Cu coatings. The reference positions of the ZrN and Cu diffractions taken from the PDF cards 00-035-0753 (ZrN) and 00-004-0836 (Cu) are marked with vertical dashed lines. A shift of ZrN peaks to higher diffraction angles is evident with increasing Cu content, which can be explained by the partial substitution of Zr by Cu atoms, as Cu has a smaller atomic radius than Zr [42]. ZrN crystallite size did not change with an increase in Cu content (the crystallite size was calculated to be ~9.7 nm for Cu11 and ~9.2 nm for Cu25). Cu11 exhibited a very weak Cu peak, corresponding with the (111) reflection. Cu25 showed a distinct Cu(111) peak of higher intensity and the emergence of a weak Cu(200) reflection. The Cu crystallite size in Cu25 was calculated to be ~5 nm. This indicates that increasing the Cu content of the coating led to the growth of the Cu phase. Hence, Cu atoms lay in the ZrN lattice and grain boundaries simultaneously [37]. 

### 3.2. Long-Term Bactericidal Properties

#### 3.2.1. Coupons

The antibacterial properties of copper are not new and have been widely studied and confirmed by various researchers [11,26,27,43,44,45,46]. The main objective of this work, however, was to determine whether copper can retain its bactericidal potency for a prolonged period of time under actual operational conditions.

The evolution of bactericidal performance of the ZrN-Cu coatings against *E. coli* CCM 3988 was assessed by two groups of samples: one group (Cu11 and Cu25) was attached onto door handles to be touched in real conditions, and another group (Cu11 and Cu25) was kept untouched to examine the aging effect on the samples’ bactericidal properties. The same testing procedure described in Section 2.3 was applied to both groups.

**Figure 3:**
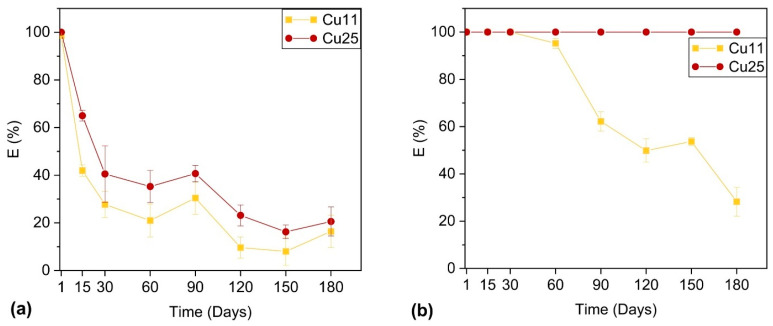
Bactericidal efficiency vs. time for (**a**) constantly touched and (**b**) untouched ZrN-Cu coatings against *E. coli* CCM. Exposure time was 40 min.

Figure 3 compares the bactericidal efficiency of Cu11 and Cu25 coatings under touched and untouched conditions. As-deposited samples showed 100% bactericidal efficiency against *E. coli* CCM 3988. Figure 3a shows a significant decline in the bactericidal activity of ZrN-Cu coatings after 15 days of touching: it drops to 65% for Cu25 and 42% for Cu11. After the touching of the samples for 1 month, the bactericidal efficiency decreased even further, dropping to 40% for Cu25 and 28% for Cu11. It appears that in the first 30 days, bactericidal efficiency declined very quickly. Even though extending the touching period led to a further fall in the bactericidal performance of the coatings, its rate was slower than in the first 30 days and, in the following 5 months, the bactericidal efficiency only decreased to 20% for Cu25 and 16% for Cu11. The untouched samples performed much better in terms of bactericidal effectiveness (Figure 3b). The effectiveness of Cu11 began to deteriorate after two months, even without being touched. However, Cu25 maintained a 100% bactericidal efficiency even after 6 months (the whole length of this study).

**Figure 4:**
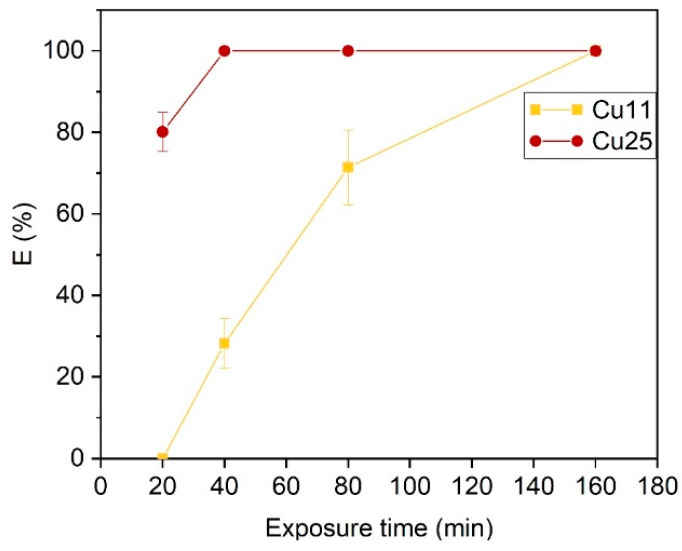
Bactericidal behavior of 6-month stored (untouched) ZrN-Cu samples at various exposure times.

The bactericidal efficiency of the ZrN-Cu coatings was further evaluated after 6 months at the end of the study by exposing them to *E. coli* CCM 3988 for different durations: 20, 40, 80, and 160 min. The touched coatings were not tested due to an insufficient number of samples. However, the untouched coatings exhibited a consistent behavior, as shown in Figure 4. This figure reveals that the aged untouched Cu11 coatings still retained some bactericidal activity, but it was slower than that of the as-deposited samples. For instance, the bactericidal efficacy of untouched Cu11 was only ~30% after 40 min of exposure, but it reached 100% after 160 min. As mentioned earlier, Cu25 remained highly effective even after several months of aging. It is worth noting that the ISO 22196 standard procedure to evaluate the bactericidal ability of surfaces proposes a bacterial exposure time of 24 h [38]. Therefore, naturally aged Cu11 can still be considered a bactericidal coating. 

**Figure 5:**
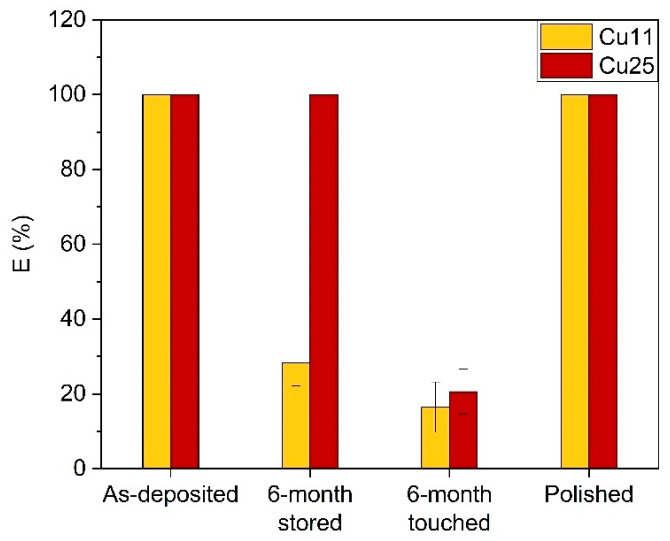
Bactericidal efficiency of polished Cu11 and Cu25 samples against *E. coli* CCM 3988. The results of as-deposited, stored, and touched samples are plotted for comparison. Bacterial exposure time was 40 min for all samples.

To maintain the bactericidal effectiveness of the coatings, it is necessary to restore it when it declines. One practical solution is to polish the surface of the samples, which can renew the surface and improve its bactericidal activity. To test this, Cu11 and Cu25 samples were polished after being touched for 6 months, and their bactericidal effectiveness was evaluated. The results showed that both samples exhibited 100% bactericidal effectiveness after polishing, as shown in Figure 5. This graph summarizes the findings of this section and clearly demonstrates that human touch can cause deterioration in a surface’s bactericidal ability, which can be restored through polishing. Additionally, the graph indicates that the as-deposited coatings had 100% efficiency, but merely storing the coatings without any touch could reduce their effectiveness, in the case of the Cu11 sample. It appears that polishing shaves off the surface layer where the Cu was depleted, allowing copper to be released again.

#### 3.2.2. Cylindrical Samples

**Figure 6:**
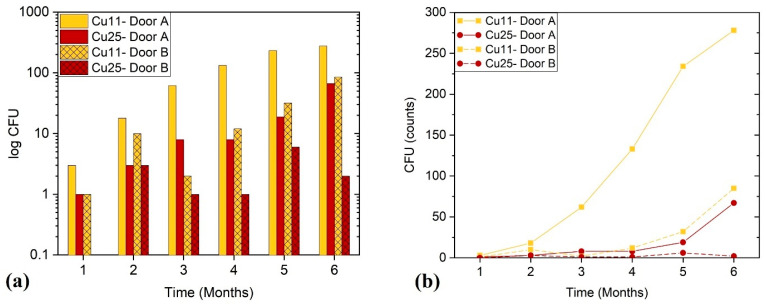
The number of viable naturally occurring bacteria in (**a**) logarithmic scale and (**b**) linear scale vs. time for ZrN-Cu coatings on the door handles installed at CCM. It should be noted that the CFU count on Cu25-Door B after 1 month was 0, so the corresponding bar is missing in (**a**).

The main objective of this part of the study was to assess the ability of the ZrN-Cu coatings to inhibit different species of bacteria on door handles that originate from human hands and the surrounding environment. No specific strain was tested in this part of the study, as stated in Section 2.3. Figure 6 shows the total number of natural and viable bacteria on the investigated door handles. Door A was touched more frequently than door B (about 3–4 times more), which resulted in higher CFU counts in this figure. Both linear and logarithmic scales were used to plot the results for better visual clarity. Cu25 showed better bactericidal ability compared to Cu11, as is indicated by the lower number of viable bacteria in Figure 6. Furthermore, this figure indicates a fall in bactericidal effectiveness over time due to human touch. Both aforementioned findings are consistent with the coupons’ results presented in Section 3.2.1.

The exact mechanism of how copper causes bacterial death is still debated in the scientific literature. There are two main hypotheses that have been proposed and supported by experimental works: (1) The first hypothesis suggests that copper damages the cell membranes of bacteria, which leads to cell lysis and death. DNA damage is a secondary effect that occurs after the cell membrane is disrupted. (2) The second hypothesis proposes that copper induces genotoxicity, which means that it harms the DNA of bacteria and prevents it from replicating. Cell membrane damage is a consequence of DNA damage and cell death [27].

The reasons for the significant change in the bactericidal performance of the coatings over time needed to be clarified. Therefore, various analyses and measurements were performed on the touched and untouched samples. The results will be presented in the following section.

### 3.3. Post-Exposure Analyses

#### 3.3.1. Roughness

The impact of surface roughness on the antimicrobial capabilities was studied, as it is a critical factor that affects bacterial adhesion and elimination [36,47,48,49]. The *S_a_* values for as-deposited, untouched, and touched samples are shown in Table 3. The findings indicate that the roughness of Cu25 is consistently greater than that of Cu11. Furthermore, there is no notable distinction between the roughness values of the as-deposited and touched samples, indicating that the deterioration of bactericidal efficiency was not due to surface smoothing or roughening.

#### 3.3.2. Microstructure

**Figure 7:**
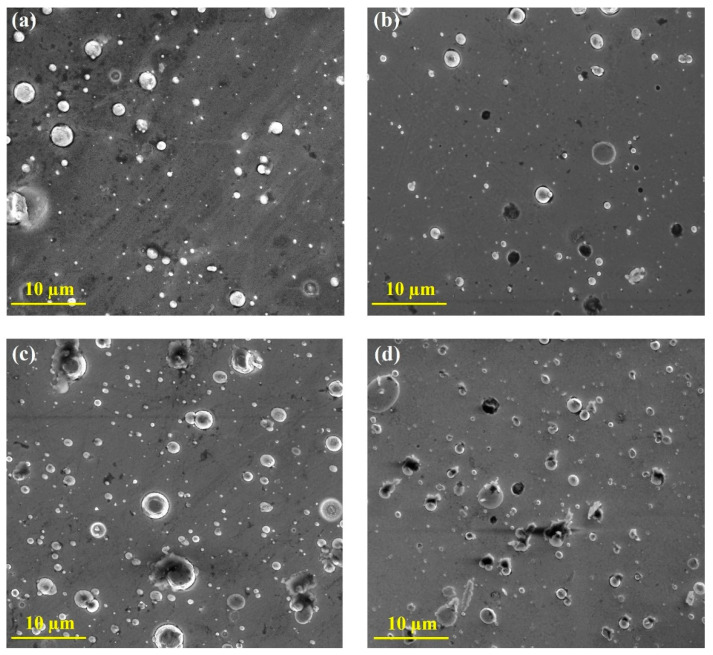
SEM micrographs of as-deposited Cu11 (**a**), touched Cu11 (**b**), as-deposited Cu25 (**c**), and touched Cu25 (**d**).

Figure 7 displays the SEM images of the as-deposited and touched coatings. These images reveal a large number of microparticles on the surface of the ZrN-Cu samples. These particles are the typical defects associated with cathodic arc evaporation and are weakly attached to the surface of the coating [50]. After being touched for 6 months, some of the microparticles were removed from the surface, as is shown in Figure 7. The SEM micrographs of the as-deposited and untouched samples did not show any remarkable changes in the surface morphology. Because the untouched samples’ surfaces were similar to that of the as-deposited ones, the corresponding images have not been presented here. The SEM images of the untouched samples can be found in the Supplementary Materials as Figure S1.

#### 3.3.3. Cu Release Rate

Previous studies have shown that the bactericidal properties of copper are attributed to its ability to release Cu ions, which can damage cell membranes [13,26]. It has been reported that higher levels of copper in Ti-6Al-4V alloy result in greater release of Cu ions, leading to stronger antibacterial effects [51]. Liu et al. [52] also found that the number of ions released from Ti-Cu alloy increased with bacterial exposure time, resulting in improved antibacterial performance. Therefore, a Cu release test was conducted to investigate the decline in bactericidal efficacy of ZrN-Cu after being touched for several months.

**Figure 8:**
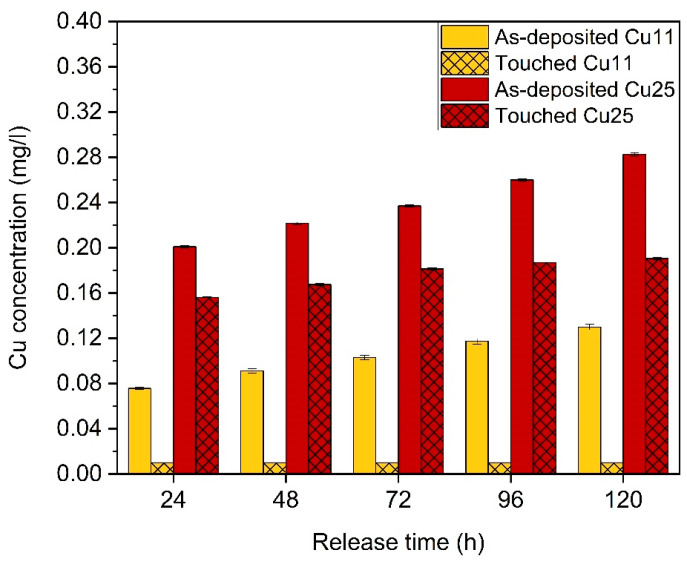
Cumulative Cu ion release profiles from ZrN-Cu samples in Milli-Q water.

The Cu release rate from the surface of the ZrN-Cu coatings was measured for the as-deposited and touched samples, both Cu11 and Cu25. Figure 8 shows the cumulative release of Cu ions from the Cu11 and Cu25 coatings. A rapid Cu ion release rate was observed at the first 24 h immersion. Afterwards, the release rate slowed down with longer immersion up to 120 h. For the touched Cu11 sample, Cu was released only in the early immersion stage, and no further Cu was released after longer immersion times (48–120 h). This initial “burst” followed by a much slower release was seen in all samples, which is consistent with other work [16,53,54]. This graph clearly shows a significant drop in the ability of the coatings to release bactericide ions after functioning under high-touch conditions. Moreover, the weaker bactericidal properties of Cu11 compared to Cu25 can be attributed to the lower amount of Cu ions released.

## 4. Conclusions

ZrN-Cu coatings containing 11 at.% and 25 at.% of Cu were deposited by a PVD system combining magnetron sputtering and arc evaporation methods in a nitrogen atmosphere. The microstructure comprises Cu and ZrN phases with larger and more pronounced Cu crystallites in the higher Cu content samples. The amount of Cu significantly impacts the hardness and elastic modulus of the coatings; they both decrease with Cu content. Freshly deposited ZrN-Cu coatings completely inhibit *E. coli* CCM 3988 upon 40 min contact. The coatings containing 11 at.% Cu experience a severe decrease in their bactericidal efficiency after being stored for 6 months. Conversely, coatings containing 25 at.% Cu maintain their bactericidal effect even after being stored for 6 months. Both groups experience a severe decline in their bactericidal performance when they are exposed to the high-touch conditions on door handles. 

In the cylindrical samples, more-frequently touched door handles exhibit higher CFU counts. Cu25 shows better bactericidal ability compared to Cu11. Similarly to the coupons, bactericidal efficiency is found to decline over time due to human touch.

The Cu release rate from the coatings’ surface decreases with touching, which may be responsible for the drop in the bactericidal ability of the coatings. Polishing the samples was found to be a practical solution for restoring their bactericidal properties. Considering the good mechanical and bactericidal properties of ZrN-Cu, these coatings have the potential to be applied on high-touch surfaces to prevent the spread of bacterial infections. A potential limitation of ZrN-Cu coatings is the gradual loss of their bactericidal efficiency, which requires periodic surface renewal to ensure optimal performance. Further research is recommended to explore the hybrid effects of these coatings with different cleaning methods to sustain their bactericidal activity effectively. The results of this trial clearly demonstrate that applying Cu-containing self-sanitizing coatings offers the potential to significantly reduce the numbers of bacteria and to combat the rise of antibiotic-resistant bacteria, as they can reduce the infection rate and lower the costs of hospitalization and treatment for patients.

## Figures and Tables

**Table 1:** Deposition parameters of ZrN-Cu coatings.

	Temperature (°C)	Bias Voltage (V)	N_2_ Pressure (Pa)	Arc Current (A)	Time (min)
Adhesion layer (ZrN)	400	−50	1.5	150	10
ZrN-Cu coating	400	−50	1.5	150	100

**Table 2:** Chemical composition and mechanical properties of ZrN-Cu coatings.

Sample	Cu (at.%)	Zr (at.%)	N (at.%)	H (GPa)	E (GPa)
Cu11	11	45.7	43.3	24.2 ± 0.7	299 ± 8
Cu25	25.1	37.9	37	17.8 ± 0.3	242 ± 7

**Table 3:** The surface roughness values of as-deposited, untouched, and touched ZrN-Cu samples.

Sample	As-Deposited		Untouched		Touched	
	Cu11	Cu25	Cu11	Cu25	Cu11	Cu25
**S_a_ (nm)**	59 ± 8	84 ± 5	60 ± 5	90 ± 8	55 ± 13	84 ± 7

## Data Availability

Data are contained within the article.

## Supplementary Materials

The following supporting information can be downloaded at: https://www.mdpi.com/article/10.3390/nano14060496/s1. SEM micrographs of the untouched samples.

## References

[1] Mohammady M., Radmeh M., Taherian H., Tahmourespour A., Hashemi S.T. (2021). Effect of Copper-Coated Surfaces on the Bacterial Burden in the Intensive Care Unit. Iran. Red Crescent Med. J..

[2] Hinsa-Leasure S.M., Nartey Q., Vaverka J., Schmidt M.G. (2016). Copper Alloy Surfaces Sustain Terminal Cleaning Levels in a Rural Hospital. Am. J. Infect. Control.

[3] Bisht N., Dwivedi N., Kumar P., Venkatesh M., Yadav A.K., Mishra D., Solanki P., Verma N.K., Lakshminarayanan R., Ramakrishna S. (2022). Recent Advances in Copper and Copper-Derived Materials for Antimicrobial Resistance and Infection Control. Curr. Opin. Biomed. Eng..

[4] Of S., In H., Eu T.H.E. (2018). Health at a Glance: Europe 2018 (Summary in Spanish).

[5] Abraham J., Dowling K., Florentine S. (2021). Can Copper Products and Surfaces Reduce the Spread of Infectious Microorganisms and Hospital-Acquired Infections?. Materials.

[6] Potter B.A., Lob M., Mercaldo R., Hetzler A., Kaistha V., Khan H., Kingston N., Knoll M., Maloy-Franklin B., Melvin K. (2015). A Long-Term Study Examining the Antibacterial Effectiveness of Agion Silver Zeolite Technology on Door Handles within a College Campus. Lett. Appl. Microbiol..

[7] Aboubakr H.A., Sharafeldin T.A., Goyal S.M. (2021). Stability of SARS-CoV-2 and Other Coronaviruses in the Environment and on Common Touch Surfaces and the Influence of Climatic Conditions: A Review. Transbound. Emerg. Dis..

[8] Bueckert M., Gupta R., Gupta A., Garg M., Mazumder A. (2021). Correction: Bueckert et al. Infectivity of SARS-CoV-2 and Other Coronaviruses on Dry Surfaces: Potential for Indirect Transmission. *Materials* 2020, *13*, 5211. Materials.

[9] Glass A., Klinkhammer K.E., Christofferson R.C., Mores C.N. (2022). Efficacy of Copper Blend Coatings in Reducing SARS-CoV-2 Contamination. BioMetals.

[10] Bäumler W., Eckl D., Holzmann T., Schneider-Brachert W. (2022). Antimicrobial Coatings for Environmental Surfaces in Hospitals: A Potential New Pillar for Prevention Strategies in Hygiene. Crit. Rev. Microbiol..

[11] Nakhaie D., Williams T.C., Velapatino B., Bryce E.A., Charles M.K., Asselin E., Clifford A.M. (2022). An Engineered Nanocomposite Copper Coating with Enhanced Antibacterial Efficacy. Adv. Mater. Interfaces.

[12] Feng J., Tong S.Y., Thian E.S., Yang C., Yang K., Gong N., Misra K.P., Misra R.D.K. (2022). Nanostructuring of Biomaterials and Reducing Implant Related Infections via Incorporation of Silver and Copper as Antimicrobial Elements: An Overview. Mater. Technol..

[13] Meister T.L., Fortmann J., Breisch M., Sengstock C., Steinmann E., Köller M., Pfaender S., Ludwig A. (2022). Nanoscale Copper and Silver Thin Film Systems Display Differences in Antiviral and Antibacterial Properties. Sci. Rep..

[14] Slavin Y.N., Asnis J., Häfeli U.O., Bach H. (2017). Metal Nanoparticles: Understanding the Mechanisms behind Antibacterial Activity. J. Nanobiotechnol..

[15] Musil J., Zítek M., Fajfrlík K., Čerstvý R. (2016). Flexible Antibacterial Zr-Cu-N Thin Films Resistant to Cracking. J. Vac. Sci. Technol. A Vac. Surf. Films.

[16] Stranak V., Wulff H., Rebl H., Zietz C., Arndt K., Bogdanowicz R., Nebe B., Bader R., Podbielski A., Hubicka Z. (2011). Deposition of Thin Titanium-Copper Films with Antimicrobial Effect by Advanced Magnetron Sputtering Methods. Mater. Sci. Eng. C.

[17] Hadrup N., Sharma A.K., Loeschner K. (2018). Toxicity of Silver Ions, Metallic Silver, and Silver Nanoparticle Materials after in Vivo Dermal and Mucosal Surface Exposure: A Review. Regul. Toxicol. Pharmacol..

[18] Knobloch J.K.M., Tofern S., Kunz W., Schütze S., Riecke M., Solbach W., Wuske T. (2017). “Life-like” Assessment of Antimicrobial Surfaces by a New Touch Transfer Assay Displays Strong Superiority of a Copper Alloy Compared to Silver Containing Surfaces. PLoS ONE.

[19] Kumar D.D., Kaliaraj G.S. (2018). Multifunctional Zirconium Nitride/Copper Multilayer Coatings on Medical Grade 316L SS and Titanium Substrates for Biomedical Applications. J. Mech. Behav. Biomed. Mater..

[20] Purniawan A., Lusida M.I., Pujiyanto R.W., Nastri A.M., Permanasari A.A., Harsono A.A.H., Oktavia N.H., Wicaksono S.T., Dewantari J.R., Prasetya R.R. (2022). Synthesis and Assessment of Copper-Based Nanoparticles as a Surface Coating Agent for Antiviral Properties against SARS-CoV-2. Sci. Rep..

[21] Weaver L., Michels H.T., Keevil C.W. (2010). Potential for Preventing Spread of Fungi in Air-Conditioning Systems Constructed Using Copper Instead of Aluminium. Lett. Appl. Microbiol..

[22] Warnes S.L., Summersgill E.N., Keevil C.W. (2015). Inactivation of Murine Norovirus on a Range of Copper Alloy Surfaces Is Accompanied by Loss of Capsid Integrity. Appl. Environ. Microbiol..

[23] Warnes S.L., Keevil C.W. (2013). Inactivation of Norovirus on Dry Copper Alloy Surfaces. PLoS ONE.

[24] Govind V., Bharadwaj S., Sai Ganesh M.R., Vishnu J., Shankar K.V., Shankar B., Rajesh R. (2021). Antiviral Properties of Copper and Its Alloys to Inactivate Covid-19 Virus: A Review. BioMetals.

[25] Luo J., Hein C., Mücklich F., Solioz M. (2017). Killing of Bacteria by Copper, Cadmium, and Silver Surfaces Reveals Relevant Physicochemical Parameters. Biointerphases.

[26] Vincent M., Duval R.E., Hartemann P., Engels-Deutsch M. (2018). Contact Killing and Antimicrobial Properties of Copper. J. Appl. Microbiol..

[27] Mahmoudi P., Akbarpour M.R., Lakeh H.B., Jing F., Hadidi M.R., Akhavan B. (2022). Antibacterial Ti–Cu Implants: A Critical Review on Mechanisms of Action. Mater. Today Bio.

[28] Michels H.T., Keevil C.W., Salgado C.D., Schmidt M.G. (2015). From Laboratory Research to a Clinical Trial: Copper Alloy Surfaces Kill Bacteria and Reduce Hospital-Acquired Infections. Health Environ. Res. Des. J..

[29] Bryce E.A., Velapatino B., Donnelly-Pierce T., Wong T., Dixon R., Akbari Khorami H., Asselin E. (2022). Evaluating the Antimicrobial Activity of Copper Surfaces against Pseudomonas Aeruginosa and Staphylococcus Aureus 1 Year after Use in a Microbiology Laboratory. J. Hosp. Infect..

[30] Casey A.L., Adams D., Karpanen T.J., Lambert P.A., Cookson B.D., Nightingale P., Miruszenko L., Shillam R., Christian P., Elliott T.S.J. (2010). Role of Copper in Reducing Hospital Environment Contamination. J. Hosp. Infect..

[31] Mikolay A., Huggett S., Tikana L., Grass G., Braun J., Nies D.H. (2010). Survival of Bacteria on Metallic Copper Surfaces in a Hospital Trial. Appl. Microbiol. Biotechnol..

[32] Ji M.K., Park S.W., Lee K., Kang I.C., Yun K.D., Kim H.S., Lim H.P. (2015). Evaluation of Antibacterial Activity and Osteoblast-like Cell Viability of TiN, ZrN and (Ti1-XZrx)N Coating on Titanium. J. Adv. Prosthodont..

[33] Brunello G., Brun P., Gardin C., Ferroni L., Bressan E., Meneghello R., Zavan B., Sivolella S. (2018). Biocompatibility and Antibacterial Properties of Zirconium Nitride Coating on Titanium Abutments: An in Vitro Study. PLoS ONE.

[34] Ramoul C., Beliardouh N.E., Bahi R., Nouveau C., Djahoudi A., Walock M.J. (2019). Surface Performances of PVD ZrN Coatings in Biological Environments. Tribol.—Mater. Surf. Interfaces.

[35] Tian X.B., Wang Z.M., Yang S.Q., Luo Z.J., Fu R.K.Y., Chu P.K. (2007). Antibacterial Copper-Containing Titanium Nitride Films Produced by Dual Magnetron Sputtering. Surf. Coat. Technol..

[36] Sharifahmadian O., Salimijazi H.R., Fathi M.H., Mostaghimi J., Pershin L. (2013). Relationship between Surface Properties and Antibacterial Behavior of Wire Arc Spray Copper Coatings. Surf. Coat. Technol..

[37] Behrangi S., Sedláček I., Štěrba J., Suková G., Czigány Z., Buršíková V., Souček P., Sochora V., Balázsi K., Vašina P. (2022). An Assessment of the Bactericidal and Virucidal Properties of ZrN-Cu Nanostructured Coatings Deposited by an Industrial PVD System. Coatings.

[38] (2011). Measurement of Antibacterial Activity on Plastics and Other Non-Porous Surfaces.

[39] Cullity B.D., Stock S.R. (2013). Elements of X-Ray Diffraction: Pearson New International.

[40] Oliver W.C., Pharr G.M. (1992). An Improved Technique for Determining Hardness and Elastic Modulus Using Load and Displacement Sensing Indentation Experiments. J. Mater. Res..

[41] Ciacotich N., Din R.U., Sloth J.J., Møller P., Gram L. (2018). An Electroplated Copper–Silver Alloy as Antibacterial Coating on Stainless Steel. Surf. Coat. Technol..

[42] Slater J.C. (1964). Atomic Radii in Crystals. J. Chem. Phys..

[43] Li X., Cong Y., Ovais M., Cardoso M.B., Hameed S., Chen R., Chen M., Wang L. (2023). Copper-Based Nanoparticles against Microbial Infections. Wiley Interdiscip. Rev. Nanomed. Nanobiotechnol..

[44] Seo B., Kanematsu H., Nakamoto M., Miyabayashi Y., Tanaka T. (2022). Copper Surface Treatment Method with Antibacterial Performance Using “Super-Spread Wetting” Properties. Materials.

[45] Robine E., Boulangé-Petermann L., Derangère D. (2002). Assessing Bactericidal Properties of Materials: The Case of Metallic Surfaces in Contact with Air. J. Microbiol. Methods.

[46] Dan Z.G., Ni H.W., Xu B.F., Xiong J., Xiong P.Y. (2005). Microstructure and Antibacterial Properties of AISI 420 Stainless Steel Implanted by Copper Ions. Thin Solid Films.

[47] Ishantha Senevirathne S.W.M.A., Hasan J., Mathew A., Jaggessar A., Yarlagadda P.K.D.V. (2021). Trends in Bactericidal Nanostructured Surfaces: An Analytical Perspective. ACS Appl. Bio Mater..

[48] Hutasoit N., Topa S.H., Javed M.A., Rahman Rashid R.A., Palombo E., Palanisamy S. (2021). Antibacterial Efficacy of Cold-Sprayed Copper Coatings against Gram-Positive Staphylococcus Aureus and Gram-Negative *Escherichia coli*. Materials.

[49] Razavipour M., Gonzalez M., Singh N., Cimenci C.E., Chu N., Alarcon E.I., Villafuerte J., Jodoin B. (2022). Biofilm Inhibition and Antiviral Response of Cold Sprayed and Shot Peened Copper Surfaces: Effect of Surface Morphology and Microstructure. J. Therm. Spray Technol..

[50] Gilewicz A., Jedrzejewski R., Myslinski P., Warcholinski B. (2019). Structure, Morphology, and Mechanical Properties of AlCrN Coatings Deposited by Cathodic Arc Evaporation. J. Mater. Eng. Perform..

[51] Ren L., Ma Z., Li M., Zhang Y., Liu W., Liao Z., Yang K. (2014). Antibacterial Properties of Ti-6Al-4V-XCu Alloys. J. Mater. Sci. Technol..

[52] Liu J., Zhang X., Wang H., Li F., Li M., Yang K., Zhang E. (2014). The Antibacterial Properties and Biocompatibility of a Ti-Cu Sintered Alloy for Biomedical Application. Biomed. Mater..

[53] Unosson E., Rodriguez D., Welch K., Engqvist H. (2015). Reactive Combinatorial Synthesis and Characterization of a Gradient Ag-Ti Oxide Thin Film with Antibacterial Properties. Acta Biomater..

[54] Wang S., Zhu W., Yu P., Wang X., He T., Tan G., Ning C. (2015). Antibacterial Nanostructured Copper Coatings Deposited on Tantalum by Magnetron Sputtering. Mater. Technol..

