# Supplementary figures and images for: A Long-Term Study on the Bactericidal Effect of ZrN-Cu Nanostructured Coatings Deposited by an Industrial Physical Vapor Deposition System
Source: Nanomaterials (Basel). 2024 Mar 10;14(6):496. doi: 10.3390/nano14060496 (PMC10975799; doi:10.3390/nano14060496)

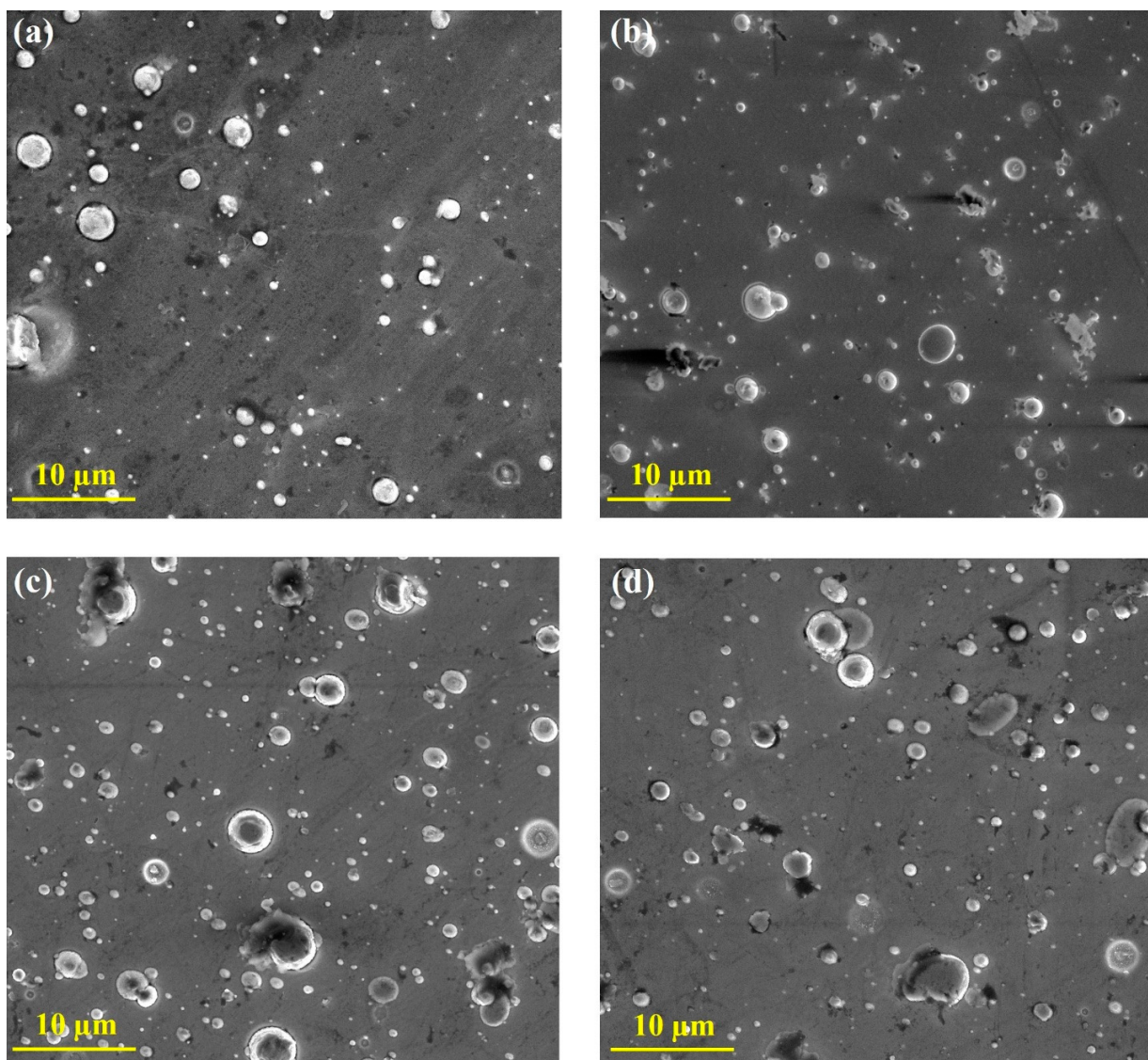

**Fig. S1.** SEM micrographs of as-deposited Cu11 (a), untouched Cu11 (b), as-deposited Cu25 (c) and untouched Cu25 (d)..

Supplement: Supplementary file 1 [file nanomaterials-14-00496-s001.zip › nanomaterials-2887307-supplementary.pdf]
